# Oral streptococci *S. anginosus* and *S. mitis* induce distinct morphological, inflammatory, and metabolic signatures in macrophages

**DOI:** 10.1128/iai.00536-23

**Published:** 2024-01-30

**Authors:** Sangeetha Senthil Kumar, Venugopal Gunda, Dakota M. Reinartz, Kelvin W. Pond, Curtis A. Thorne, Paul Victor Santiago Raj, Michael D. L. Johnson, Justin E. Wilson

**Affiliations:** 1 Department of Immunobiology, College of Medicine, The University of Arizona, Tucson, Arizona, USA; 2 The University of Arizona Cancer Center, Tucson, Arizona, USA; 3 Department of Pharmacology, University of Virginia, Charlottesville, Virginia, USA; 4 Department of Cellular and Molecular Medicine, The University of Arizona, Tucson, Arizona, USA; 5 R. Ken Coit College of Pharmacy, The University of Arizona, Tucson, Arizona, USA; 6 Valley Fever Center for Excellence, The University of Arizona Health Sciences, Tucson, Arizona, USA; 7 BIO5 Institute, The University of Arizona, Tucson, Arizona, USA; 8 Asthma and Airway Disease Research Center, The University of Arizona Health Sciences, Tucson, Arizona, USA; University of Pennsylvania Perelman School of Medicine, Philadelphia, Philadelphia, USA

**Keywords:** oral streptococci, *S. anginosus*, *S. mitis*, inflammation, macrophages, immunometabolism

## Abstract

Oral streptococci, key players in oral biofilm formation, are implicated in oral dysbiosis and various clinical conditions, including dental caries, gingivitis, periodontal disease, and oral cancer. Specifically, *Streptococcus anginosus* is associated with esophageal, gastric, and pharyngeal cancers, while *Streptococcus mitis* is linked to oral cancer. However, no study has investigated the mechanistic links between these *Streptococcus* species and cancer-related inflammatory responses. As an initial step, we probed the innate immune response triggered by *S. anginosus* and *S. mitis* in RAW264.7 macrophages. These bacteria exerted time- and dose-dependent effects on macrophage morphology without affecting cell viability. Compared with untreated macrophages, macrophages infected with *S. anginosus* exhibited a robust proinflammatory response characterized by significantly increased levels of inflammatory cytokines and mediators, including TNF, IL-6, IL-1β, NOS2, and COX2, accompanied by enhanced NF-κB activation. In contrast, *S. mitis*-infected macrophages failed to elicit a robust inflammatory response. Seahorse Xfe96 analysis revealed an increased extracellular acidification rate in macrophages infected with *S. anginosus* compared with *S. mitis*. At the 24-h time point, the presence of *S. anginosus* led to reduced extracellular itaconate, while *S. mitis* triggered increased itaconate levels, highlighting distinct metabolic profiles in macrophages during infection in contrast to aconitate decarboxylase expression observed at the 6-h time point. This initial investigation highlights how *S. anginosus* and *S. mitis*, two Gram-positive bacteria from the same genus, can prompt distinct immune responses and metabolic shifts in macrophages during infection.

**IMPORTANCE:**

The surge in head and neck cancer cases among individuals devoid of typical risk factors such as Human Papilloma Virus (HPV) infection and tobacco and alcohol use sparks an argumentative discussion around the emerging role of oral microbiota as a novel risk factor in oral squamous cell carcinoma (OSCC). While substantial research has dissected the gut microbiome’s influence on physiology, the oral microbiome, notably oral streptococci, has been underappreciated during mucosal immunopathogenesis. *Streptococcus anginosus*, a viridans streptococci group, has been linked to abscess formation and an elevated presence in esophageal cancer and OSCC. The current study aims to probe the innate immune response to *S. anginosus* compared with the early colonizer *Streptococcus mitis* as an important first step toward understanding the impact of distinct oral *Streptococcus* species on the host immune response, which is an understudied determinant of OSCC development and progression.

## INTRODUCTION

Microbes that are in constant interaction with the host engage in varied interactions spanning symbiosis, and the outcomes range from damage, benefit, or indifference (1). The delicate interaction between the body’s resident microbiota and the immune system is vital for maintaining homeostatic balance. Disruption in this immune homeostasis due to dysbiosis can be elicited by various lifestyle factors like tobacco consumption, chronic alcohol exposure, inadequate oral hygiene, dietary changes, or antibiotic usage. While host damage is often a requirement for the induction of a pathogen-specific immune response, the extent and nature of the damage incurred during the early stages and/or subsequent phases between host and microbial interactions determine the outcome of the host-microbe relationship (2). The occurrence of damage isn’t solely attributed to the presence and proliferation of pathogens; rather, it can also stem from the deficiency of commensal bacteria that typically inhibit the colonization of these pathogens.

Oral commensal streptococci or viridans group streptococci (VGS) are Gram-positive cocci that are catalase-negative facultative anaerobes. While most exhibit alpha hemolysis, variations exist, with some demonstrating beta hemolysis or displaying no hemolytic activity when cultured on blood agar (3). Streptococci are the first organisms to colonize oral surfaces, and most of them are typically deemed non-pathogenic due to their lack of virulence factors, like exotoxin production, and due to their non-invasive potential found in other pathogenic organisms. Being early colonizers of the oral cavity, *Streptococcus* species like *Streptococcus mitis*, *Streptococcus anginosus*, *Streptococcus gordonii*, *Streptococcus oralis*, and *Streptococcus salivarius* (4) play a significant role in maintaining oral homeostasis by establishing a stable multispecies dental biofilm (5). Despite being traditionally considered non-pathogenic, they can also become opportunistic pathogens, evidenced by their involvement in a diverse range of clinical diseases, including infective endocarditis (6), bacteremia (7), and infections related to malignancy (8).

The *Streptococcus anginosus* group (SAG), formerly known as the *Streptococcus milleri* group, refers to a subgroup of the VGS composed of three species, namely, *S. anginosus*, *Streptococcus constellatus*, and *Streptococcus intermedius*. Although SAG bacteria were first detected in dental abscesses, leading to oral infections, their pathogenicity has extended beyond oral cavities, and they are now increasingly identified as causative agents of infections in various anatomical locations, exhibiting species-specific clinical characteristics (9, 10). Systemic *S. anginosus* infections have been reported in patients with esophageal, oral, and colorectal cancer (11, 12). Furthermore, *S. anginosus* is considered as a relevant marker of head, neck, and esophageal cancers and is more common in oral squamous cell carcinoma (13, 14).

*S. mitis*, another commensal and one of the early colonizers of the oral cavity, is also reported to be significantly elevated in the saliva of patients with oral squamous cell carcinoma with a diagnostic sensitivity and specificity of 80% and 82%, respectively (15). In addition, *S. mitis* is reportedly involved in bacteremia and endocarditis (16). A retrospective study conducted by Su et al. (17) identified the *S. anginosus* group (38.8%) and *S. mitis* group (22.8%) as the most common oral streptococci species present in adult patients with a monomicrobial blood culture positive for oral streptococci. Studies have shown that the abundance of saccharolytic and aciduric species, including *S. mitis*, is considerably higher on tumor surfaces than on non-tumor tissue (18). Although a higher prevalence of *S. mitis* and *S. anginosus* has been observed in cancerous esophageal tissue (19), the potential implications of this microbial enrichment on the carcinogenic process remain unclear and warrant further investigation (20). Understanding the host response to these bacteria may provide insights to identify the role of these streptococci species in carcinogenesis.

Macrophages act as tissue sentinels of the immune system, detecting microbes via Pattern Recognition Receptors and cytokine signals, resulting in the development of M1 and M2 phenotypes. M1 macrophages are activated by interferon gamma and pathogen-associated molecular patterns (PAMPs) like lipopolysaccharide (LPS) and peptidoglycan. They excel in generating reactive oxygen and nitrogen species along with type 1 proinflammatory cytokines such as TNF, IL-6, and IL-1β, which are crucial for initiating robust immune responses (21). M1 macrophages are identified by surface markers like CD80, CD86, and CD16/32. Conversely, M2 macrophages are activated by IL-4 and IL-13 and can dampen type 1 inflammation while facilitating wound healing. M2 macrophages are characterized by markers like arginase-1, CD206, and production of the anti-inflammatory cytokine IL-10. They also respond to helminth infections and allergies by producing chemokines CCL17 and CCL22 (22). In bacterial infections, macrophages usually increase the expression of genes related to M1 polarization. This pattern signifies a robust activation that acts as a general alarm against bacteria. Interestingly, these genes are often triggered regardless of the specific bacterial species and offer protection during acute infectious diseases (23). In this study, we investigated the morphological, inflammatory, and metabolic attributes of the murine macrophage RAW264.7 cells upon exposure to S. *anginosus* and *S. mitis* infections.

## MATERIALS AND METHODS

### Bacteria, chemicals, and reagents

*Streptococcus anginosus* (ATCC 33397) and *Streptococcus mitis* (ATCC 49456) and murine RAW 264.7 macrophages (TIB-71) were procured from the American Type Culture Collection (Manassas, VA, USA). BD BACTO Brain Heart Infusion and tryptic soy agar were acquired from Fischer Scientific, PA, USA. The culture medium used was Dulbecco’s modified Eagle’s medium (DMEM) supplemented with glutamine and sodium pyruvate (Corning Life Science, NY, USA). For gene expression analysis of cytokines and inflammatory mediators, namely, TNF (Mm0043258), IL-6 (Mm00446190), IL-1β (Mm00434228), Mrc (Mm01329362), Arg-1 (Mm00475988), IL-10 (Mm01288386) Cox2/ptgs2 (Mm00478374), and Nos2 (Mm0040502) TaqMan probes were sourced from Thermo Fisher Scientific Inc. (MA, USA). The Mouse TNFα, IL-6, and IL-1β DuoSet ELISA Kits were procured from R&D Systems (MN, USA). For protein analysis, anti-rabbit monoclonal antibodies for p65 (#8242S), phospho-p65 (#3033S), total IKBα (#4812S), phospho-IKBα (#2859S), NLRP3 (#15101S), COX2 (#12282), and Irg1/ACOD1 (17805S) were purchased from Cell Signaling Technology (Danvers, MA, USA). The anti-SDHA antibody (ab14715) and anti-iNOS antibody were procured from Abcam (Waltham, MA, USA). All other chemicals were obtained from Sigma Chemicals (St. Louis, MO, USA).

### Colony formation unit assay

Colony-forming unit (CFU) assay was performed following the protocol of Menghani et al. (24). A loop of *S. anginosus* and *S. mitis* glycerol stocks was inoculated on blood agar plates and incubated at 37°C in a 5% CO2 incubator overnight. Following this incubation, a loop of the resultant culture was removed from the blood agar plate and added to 10 mL of pre-warmed brain heart infusion broth, and the growth was monitored until the optical density at 600 nm (OD_600_) became 0.1. Upon achieving the desired OD600, serial dilutions of the broth culture were prepared from 100 µL of the stock culture in 96-well plates, with 10 µL of each dilution plated on the blood agar (*n* = 6). The plates were then incubated for 24 h in a 5% CO2 incubator at 37°C. The CFU were then enumerated to determine the viable cell count (Fig. S1).

### Bacteria and macrophage coculture

RAW 264.7 macrophages were grown as an adherent monolayer culture in 1× DMEM supplemented with 10% fetal bovine serum (FBS) and 5% penicillin-streptomycin. Cells were maintained in humidified air containing 5% CO2 at 37°C overnight. The next day, the media were removed, cells were washed with PBS, and DMEM medium with FBS devoid of antibiotics was added just prior to bacterial infection. To prepare bacterial inoculants, a loop of glycerol stock obtained from *S. anginosus* and *S. mitis* was streaked on a blood agar plate and incubated at 37°C in a 5% CO2 incubator for overnight growth. The next day, a loop of bacteria grown overnight on the blood agar plate was scraped and inoculated in brain heart infusion broth at 37°C with 5% CO2 and allowed to grow until an OD600 of 0.1. Then, the appropriate amount of culture corresponding to the desired multiplicity of infection (MOI) was pipetted and pelleted by centrifuging at 4,000 rpm for 10 min at 4°C. The bacterial pellet was then resuspended in 1 mL PBS, and 100 μL of the resuspension was used to infect the macrophages with an MOI of 10. Lipopolysaccharide (LPS from *E. coli* O111:B4, InvivoGen) was used as a robust positive control for macrophage activation.

### Cell fixation in culture plates for macrophage growth and morphology

The protocol for fixing adherent cells was adapted by Martis et al. (25) with slight modifications. In brief, RAW264.7 cells were grown in 24-well plates treated with LPS, *S. anginosus*, or *S. mitis* for 6 h or 24 h with the MOI of 1, 10, or 50. After treatment, the media were removed, and the cells were washed twice with PBS. Then, 0.5 mL of 4% paraformaldehyde (PFA) was added to the cells and incubated for 15–20 min at room temperature. The PFA was aspirated, and the cells were washed twice with ice-cold PBS with a 5-min wait time between each wash. Then, 1 mL of PBS was added, and the plate was wrapped and stored at 4°C until the images were captured in an ECHO Revolve D2224.

### Crystal violet staining

Crystal violet staining was performed according to the methods of Zinser et al. (26) and Feoktistova et al. (27). The cells were fixed as described above and stored in PBS at 4°C. On the day of staining, the PBS was removed, and 200 µL of 0.25% crystal violet was added for 30 min. The stain was removed, and the plate was washed in running tap water, immersing it in an appropriate container until it became clear. The plate was then blotted on a paper towel and air dried for 15 min, and the images were captured at 20× an ECHO Revolve D2224.

### Giemsa staining

Giemsa staining was carried out by adopting the method used to quantify phagocytosis by Nicola et al. (28). RAW264.7 cell lines were plated in 96-well plates at the seeding density 5 × 10^4^. The next day, the media were removed, and the cells were infected with *S. anginosus* or *S. mitis* with DMEM media containing no antibiotics at an MOI of 10. The cells were centrifuged at 24°C for 10 min at 500 *g*. The plate was incubated at 37°C in a 5% CO2 incubator for 3 h. After the 3-h incubation, the cells were fixed with 200 µL of ice-cold methanol for 30 min. Methanol was removed, and each well was washed 2× with 200 µL of PBS followed by the 100 µL of Giemsa working solution, wrapped, and kept at 4°C overnight. After overnight incubation, the staining solution was aspirated, and the wells were washed gently with PBS. Cells were observed, and the images were captured using a Biotek citation at 40× magnification. At least 300 cells per condition were quantified using NIS-Elements image analysis software. The areas of both the cytoplasm and the nucleus were quantified manually to obtain the nucleus-to-cytoplasmic ratio.

### Cell viability and cell death assays

RAW267.4 macrophages (1.5 × 10^4 cells/well) were seeded in 100 µL of media in a 96-well black wall plate with a glass bottom for Propidium Iodide/Hoechst staining or a white wall plate for the Caspase 3/7 Glo and CCK8 assays. The following day, cells were washed with PBS and treated with LPS or bacteria in media without antibiotics and were then incubated for 24 h. After 24 h, for fluorescence microscopy, a staining solution consisting of 20 µL Hoechst (10 mg/mL stock) and 20 µL propidium iodide (0.5 mg/mL) in 5 mL of antibiotic-free media was prepared, and 100 µL of this solution was added to each well. After a 1-h incubation, images were captured using a 5% CO2-connected microscope. For the CCK8 assay, 10 µL of the CCK8 solution (Abcam Cambridge, MA, USA; Cat No. ab228554) was added and incubated for 1 h before reading absorbance at 450 nm in a microplate reader. For the Caspase-Glo 3/7 Assay, 100 µL of the glo reagent (Promega Madison, WI, USA; Cat No. G8091) was added and incubated for 1 h, followed by luminescence measurement. Staurosporine (0.2 µM) was used as a positive control for inducing cell death in all three experiments.

### RNA isolation and qPCR

Following 6 h post-infection, media were removed from the macrophages, which were then washed with PBS, and 750 μL of TRIzol was added per well of a six-well plate. The plate was sealed and stored at −80°C until the RNA isolation. RNA extraction was carried out by adopting the phenol/chloroform method published by Toni et al. (29). In brief, macrophages were scrapped in TRIzol, and lysates were then mixed and transferred to Eppendorf tubes. Chloroform was added to the lysates, which were then centrifuged at 12,000 × *g* for 15 min at 4°C. The upper phase was transferred and precipitated with isopropanol. Pellets were washed in ethanol, dried, resuspended in 20 µL nuclease-free water, and quantified using nanodrop. cDNA synthesis was performed using the iScript kit (Bio-Rad, Hercules, CA) in a final volume of 20 μL with the following temperature and time points: 25°C for 5 min, 46°C for 20 min, and 95°C for 1 min. qPCR analysis was carried out in 96-well plates using QuantStudio 3 (Eppendorf). Amplification was carried out at 95°C for 15 min and 50 cycles at 95°C for 15 s, 55°C for 30 s, and 72°C for 60 s, according to Applied Biosystems. The threshold cycle (CT), which indicates the relative abundance of the transcript, was used to quantify the gene expression using the delta Ct method normalized to GAPDH.

### Enzyme-linked immunosorbent assay

ELISA for TNFα (DY410-05), IL-6 (Dy-406-05), and IL-1β(DY401-05) were performed using the R&D systems kit per the manufacturer’s instructions, and the absorbance was read using an ELISA reader (BIO-RAD) at 450-nm and 570-nm dual filters.

### Western blot

Western blot was performed by adapting the protocol published by Wilson et al. (30) with minor modifications. In brief, total protein lysates were generated from the macrophage cultures by washing cells in PBS and then lysing the cells in ice-cold RIPA buffer containing a complete protease inhibitor and phosSTOP. The cell lysate was cleared of insoluble material by centrifugation at 14,000 × *g* for 10 min at 4°C followed by separation on SDS-polyacrylamide gel (gel % according to the protein size) and transfer to nitrocellulose membranes (Thermo Scientific, USA). After blocking with 10% non-fat dried milk in TBST (Tris buffer saline Tween-20) for 1 h, membranes were incubated with primary antibody overnight at 4°C on a gel rocker in 5% non-fat dried milk. Blots were washed with TBST and incubated with a secondary antibody for 90 min at room temperature. Membranes were rewashed three times with TBST. The membranes were incubated with ECL reagent in the dark. The membrane blots were exposed to X-ray film and processed using an automatic film processor (OPTIMAX). Band intensity was semi-quantitated using densitometric analysis (ImageJ software).

### Metabolic flux assay

Macrophage oxygen consumption (OCR) and extracellular acidification rates (ECAR) were measured using the Seahorse Extracellular Flux (XFe96) analyzer (Agilent Bioscience, US). After optimizing the seeding density through a series of experiments, RAW 264.7 cells were plated in a seahorse microculture plate (1.4 × 104 cells/well) in DMEM with FBS and antibiotics and incubated overnight for the cells to adhere. The next day, the sensor and cartridge were hydrated and kept in a CO2-free incubator at 37°C. Meanwhile, bacteria were grown, and the macrophages were infected, as described above. After 6 h post-infection, the media were removed, washed, and replaced with Seahorse XF media supplemented with glucose, pyruvate, and glutamine (Agilent) and stored in a CO2-free incubator at 37°C for 1 h. For the mitochondrial stress test, oligomycin (1.5 µM), carbonyl cyanide-4-(trifluoro ethoxy) phenylhydrazone (FCCP; 2 µM), and Rotenone/antimycin A (0.5 µM) were prepared in Mito stress assay media and loaded into the appropriate Seahorse cartridge ports. The OCR and ECAR were measured every 3 min, and the appropriate compounds were injected sequentially at 18-min intervals. ECAR and OCR were automatically calculated using the Wave software, and the graph was plotted in GraphPad Prism.

### Analysis of extracellular metabolites

Metabolite extraction for untargeted metabolomics was executed following the methodology published by Xu et al. (31). RAW264.7 cells were infected and incubated overnight with LPS, *S. anginosus* or *S. mitis*. After an overnight incubation, 1 mL of the cell culture media was collected and centrifuged at 5,000 rpm for 10 min at room temperature to pellet down bacteria and other debris. A total of 100 μL of the supernatant was added to the 900 μL of metabolite extraction solution [acetonitrile (75%), methanol (25%), and formic acid (0.2%)], which was then vortexed and centrifuged at 18,000 × *g* for 10 min at 4°C. Protein separation was performed on a Thermo Scientific Vanquish Duo UHPLC by The University of Arizona, Metabolomics Core. A Waters ACQUITY Premier HSS T3 column (1.8 µm, 2.1 mm × 150 mm) was used for reversed phase separation, and a Waters ACQUITY Premier BEH amide column (1.7 µm, 2.1 mm × 150 mm) was used for HILIC separation. For reversed phase separation, the gradient was from 99% mobile phase A (0.1% formic acid in H_2_O) to 95% mobile phase B (0.1% formic acid in methanol) over 16 min. For HILIC separation, the gradient was the same as the reversed phase with solvent A: 0.1% formic acid, 10 mM ammonium acetate, 90% acetonitrile, and 10% H_2_O, and solvent B: 0.1% formic acid, 10 mM ammonium acetate, 50% acetonitrile, and 50% H_2_O. Both columns were run at 45°C with a flow rate of 300 µL/min with an injection volume of 1 µL. Thermo Scientific Orbitrap Exploris 480 was used for data collection with a spray voltage of 3,500 V for positive mode (reverse phase separation) and 2,500 V for negative mode (HILIC separation) using the H-ESI source. The vaporizer temperature and ion transfer tube were both 350°C. Compounds were fragmented using data-dependent ms/ms with HCD collision energies of 20%, 40%, and 80%. Normalized peak areas were further analyzed using the online tool, MetaboAnalyst 5.0.

### Statistical analysis

GraphPad Prism software (version 9.4) was used for statistical analysis of the data and data visualization. One-way ANOVA followed by Dunnet’s post hoc analysis was carried out, and all data are presented as mean ± SEM for biological triplicates.

## RESULTS

### *S. anginosus* and *S. mitis* exhibit differential effects on macrophage morphology in a time- and dose-dependent manner

RAW 264.7 macrophage cells were cultured in an antibiotic-free medium prior to infection with *S. anginosus* or *S. mitis* at different multiplicities of infection (MOI: 1, 10, and 50) for 6 h and 24 h, and macrophage growth morphology was observed using light microscopy after fixation (Fig. S2). Visualization of the fixed cells revealed significant changes in RAW264.7 cell morphology. Uninfected control cells exhibited rounded morphology, whereas 6 h of LPS treatment resulted in typical elongated spindle-like macrophage morphology presented during activation. Even greater elongated morphology was observed in a dose- and time-dependent manner in *S. anginosus*-treated macrophages compared with LPS-treated cells (Fig. S2). In contrast, *S. mitis-*infected macrophages retained a rounded morphology at 6 h and 24 h of MOI 1 and MOI 10 treatment. However, at MOI 50, *S. mitis* induced slightly elongated morphology, although this was not to the extent of *S. anginosus* infection at any measured MOI or time point (Fig. S2). Furthermore, treatment of macrophages with LPS for 24 h resulted in mixed, distorted, dead cell-like morphology. Thus, *S. anginosus* and *S. mitis* exhibit differential effects on macrophage morphology in a time- and dose-dependent manner. Based on these morphometric findings, the MOI of 10 was chosen for further investigations.

**Fig 1:**
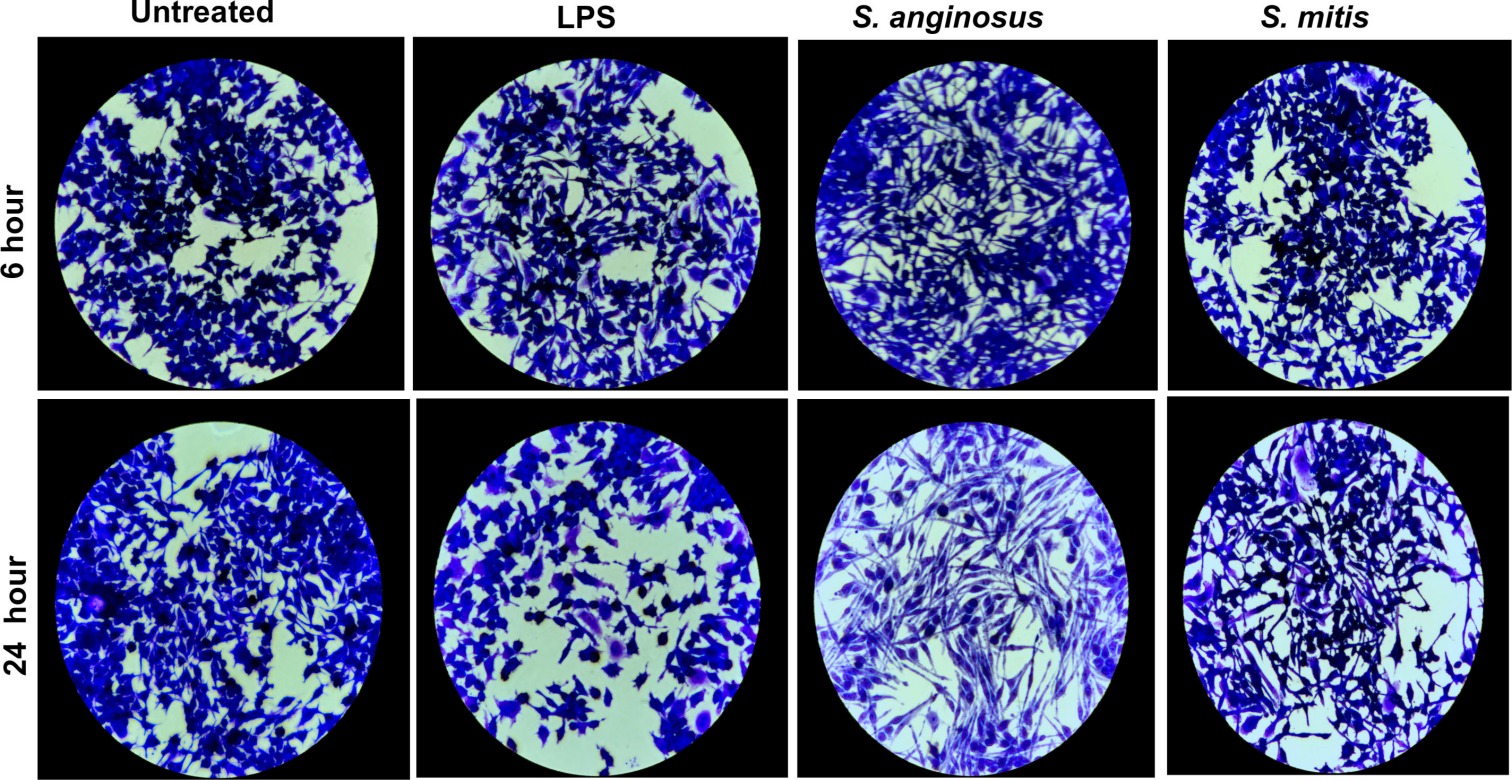
Crystal violet staining reveals distinctive RAW264.7 cell morphology in response to *S. anginosus*. Representative photograph of crystal violet staining of RAW264.7 macrophages (20×) depicts the macrophage phenotype that was analyzed after 6 h (top) and 24 h (bottom) post-infection at MOI 10. *S. anginosus*-infected macrophages showed a distinct phenotype with extended pseudopodia structures compared with untreated, LPS-treated, and *S. mitis*-infected macrophages (*n* = 3).

Light microscopy experiment was repeated with MOI 10 at 6 h and followed by crystal violet staining for greater visualization of macrophage morphology. Time-dependent induction of pseudopodia-like structures was observed in macrophages treated with *S. anginosus* and *S. mitis* compared with LPS-treated cells (Fig. 1). Again, *S. anginosus* induced more prominent spindle-like structures in macrophages after 6 h and 24 h compared with other treatment groups. These observations suggest that *S. anginosus* activates macrophages more rapidly and robustly than *S. mitis*, despite both being commensals of the oral cavity. Based on these morphometric findings, the MOI of 10 was chosen to study the innate immune response induced in RAW 264.7 macrophages by *S. anginosus* versus *S. mitis*.

### Giemsa staining reveals induction of binucleated giant macrophages during *S. anginosus* infection but not during *S. mitis* infection

**Fig 2:**
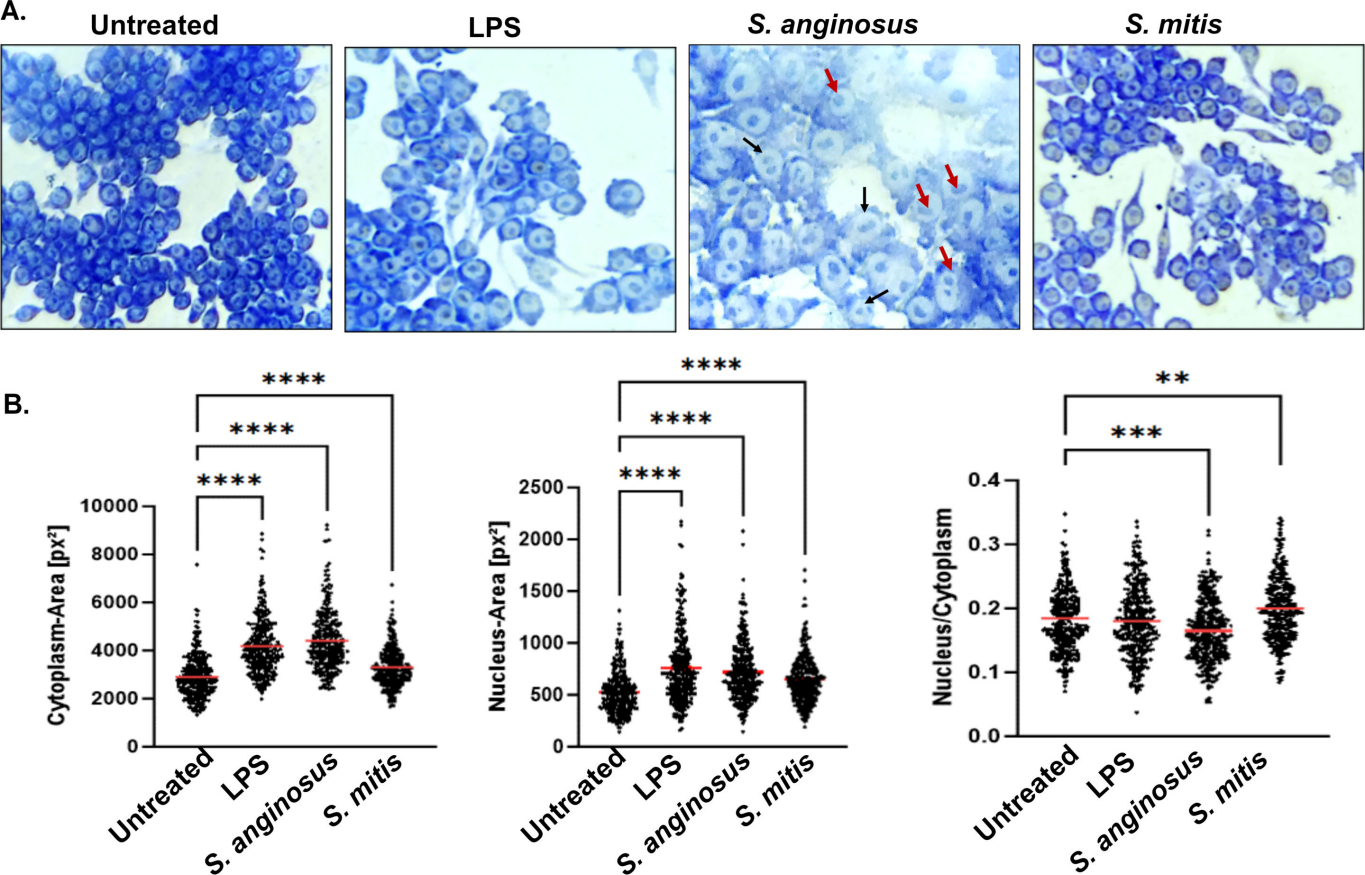
Giemsa staining showing the cytoplasm-to-nuclear ratio, highlighting the emergence of binucleated giant macrophages in *S. anginosus* infection/light microscopy images of RAW 264.7 (40×) incubated with LPS, *S. anginosus*, and *S. mitis* for 3 h revealed that S. *anginosus* infection exhibited distinct macrophage phenotype (**A**) with increased size like Giant cells, more prominent cytoplasm, and some of the nuclei are binucleated (black arrow). The peripheral position of nuclei (red arrow) is also observed in *S. anginosus*-infected macrophages. (**B**) Cell size quantification using NIS-Elements image analysis software. The nuclear to cytoplasmic ratio of activated macrophages is smaller due to expanded cytoplasm. The data in the graph are presented as mean ± SEM. *P < 0.05 and ***P < 0.0001 compared with untreated RAW 264.7 cells (*n* = 3).

Giemsa staining is a valuable tool for visualizing activated macrophages and assessing changes in cell size, as it provides contrast between cellular components, highlights morphological alterations, and facilitates quantification within macrophage-bacteria interaction studies. The present study utilized Giemsa staining to investigate the effect of *S. anginosus* and *S. mitis* on macrophage size as measured by the cytoplasm-to-nuclei ratio. When activated, macrophages are large, 20-micron-diameter cells with an elongated nucleus containing a nucleolus, a nuclear-to-cytoplasmic ratio of less than one, and a much more complex cytoplasm (32). *S. anginosus* infection resulted in distinct macrophage morphology associated with increased cellular size, more prominent cytoplasm, and the presence of binucleated nuclei (black arrow) located toward the cellular periphery (red arrow) (Fig. 2). These findings that were observed in *S. anginosus*-infected macrophages align with the morphological characteristics of activated macrophages, suggesting *S. anginosus* induces greater macrophage activation. Conversely, *S. mitis* did not prompt such pronounced morphological changes as *S. anginosus*; however, *S. mitis*-infected macrophages present significant increases in cytoplasmic and nuclear area compared with untreated macrophages. These results suggest that *S. mitis* may not induce the same level of macrophage activation as observed with *S. anginosus*.

**Fig 3:**
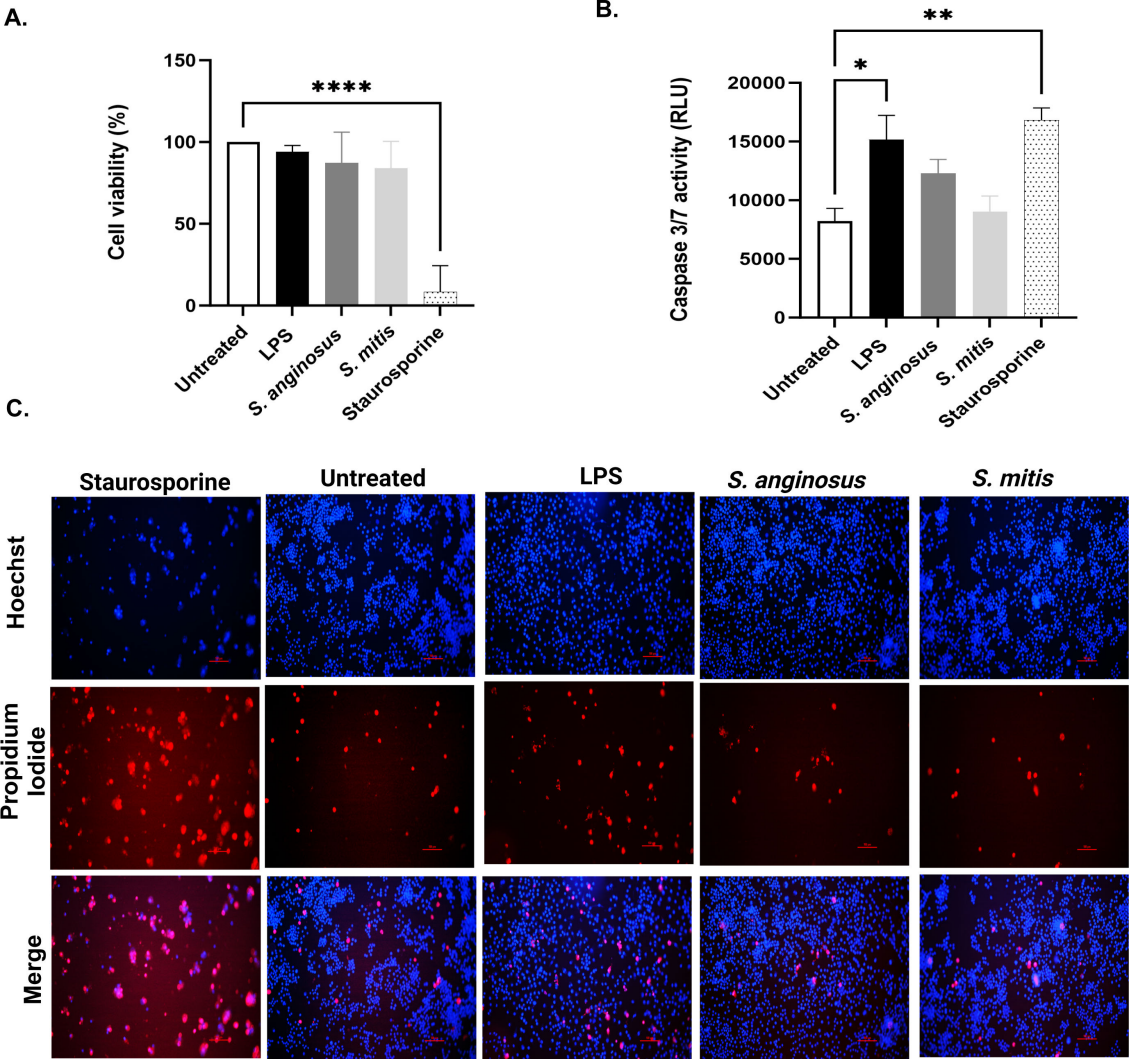
Cell viability and death assays confirm no significant cell death attributed to bacterial infection. (**A**) CCK8 assay for cell viability; (**B**) Cas 3/7 glo assay; (**C**) propidium iodide and Hoechst Live/Dead Staining. The data in the bar are presented as mean ± SEM. *P < 0.05 and ***P < 0.0001 compared with untreated RAW 264.7 cells (*n* = 3).

Because *S. anginosus* and *S. mitis* elicited distinct morphological impacts during macrophage infection, we next assessed if these infections resulted in alterations in macrophage cell survival and cell death. Following infection with *S. anginosus* or *S. mitis*, macrophage cell viability was then assessed using the cell counting (CCK8) assay, while cell death was assessed using the Caspase 3/7 Glo assay, propidium iodide staining, or Hoechst staining. These assays collectively demonstrate that neither *S. anginosus* nor *S. mitis* infection resulted in notable impacts on macrophage cellular proliferation or survival. Therefore, these results indicate that the differential effects of *S. anginosus* and *S. mitis* on macrophage morphology are not due to changes in cellular viability during these distinct infections (Fig. 3A through C)

### *S. anginosus,* but not *S. mitis,* elicits robust proinflammatory cytokine expression and production in macrophages

**Fig 4:**
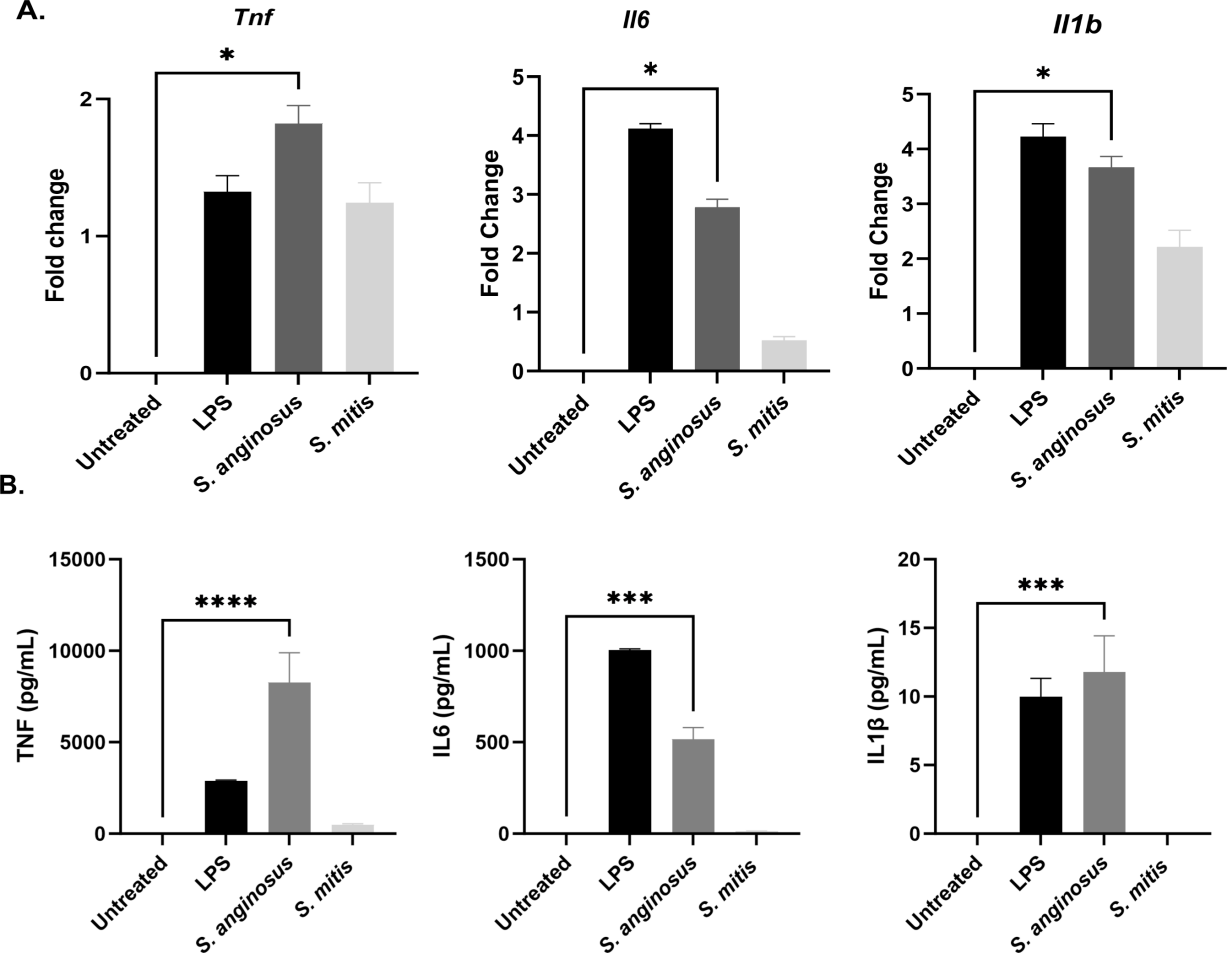
Robust induction of proinflammatory cytokines at mRNA and protein levels elicited by *S. anginosus*. (**A**) mRNA expression of cytokines from RAW 264.7 cells after 6 h infection B) Cytokine protein secretion in cell culture supernatant as measured by ELISA after 24 h post-infection. The data are presented as Mean ± SEM. *P < 0.05 and ***P < 0.0001 compared to untreated RAW 264.7 cells (*n* = 3).

Macrophages often become activated during bacterial infection, resulting in the increased expression and production of inflammatory cytokines. Because *S. anginosus* and *S. mitis* elicited distinct morphological changes in macrophages that resembled activated cells, we next investigated if these two *Streptococcus* species also elicited differential inflammatory responses. Therefore, we assessed the mRNA expression levels of the key inflammatory cytokines TNF, IL-6, and IL-1β (Fig. 4A) following 6 h post-infection with *S. anginosus* or *S. mitis* by qPCR and supernatant protein levels following 24 h of infection by ELISA (Fig. 4B). *S. anginosus*-treated macrophages displayed significantly elevated levels of proinflammatory cytokines compared to untreated macrophages. Interestingly, *S. mitis* failed to elicit this robust immune response from macrophages. Therefore, we next investigated M2 macrophage polarization and the expression of anti-inflammatory cytokines in *S. anginosus* versus *S. mitis*-infected macrophages as a potential explanation as to why *S. mitis* infection failed to elicit any observable proinflammatory responses during macrophage infection. However, qPCR analysis revealed no significant alterations in the expression of markers associated with M2 polarization, including mannose receptor (*Mrc*) or arginase (*Arg1*). Interestingly, we observed a notable increase in *Il-10* expression in *S. anginosus*-infected macrophages, however this elevation likely represents a compensatory reaction to the robust inflammatory activation observed under this infection, similar to LPS-treated controls. Importantly, we did not observe a significant induction of IL-10 or traditional M2 markers in macrophages infected with *S. mitis* (Fig. S3), arguing against M2 skewing and/or production of anti-inflammatory IL-10 as the mechanisms by which *S. mitis* fails to induce proinflammatory cytokines in macrophages.

### *S. anginosus*, but not *S. mitis*, elicits robust NF-κB activation in macrophages

**Fig 5:**
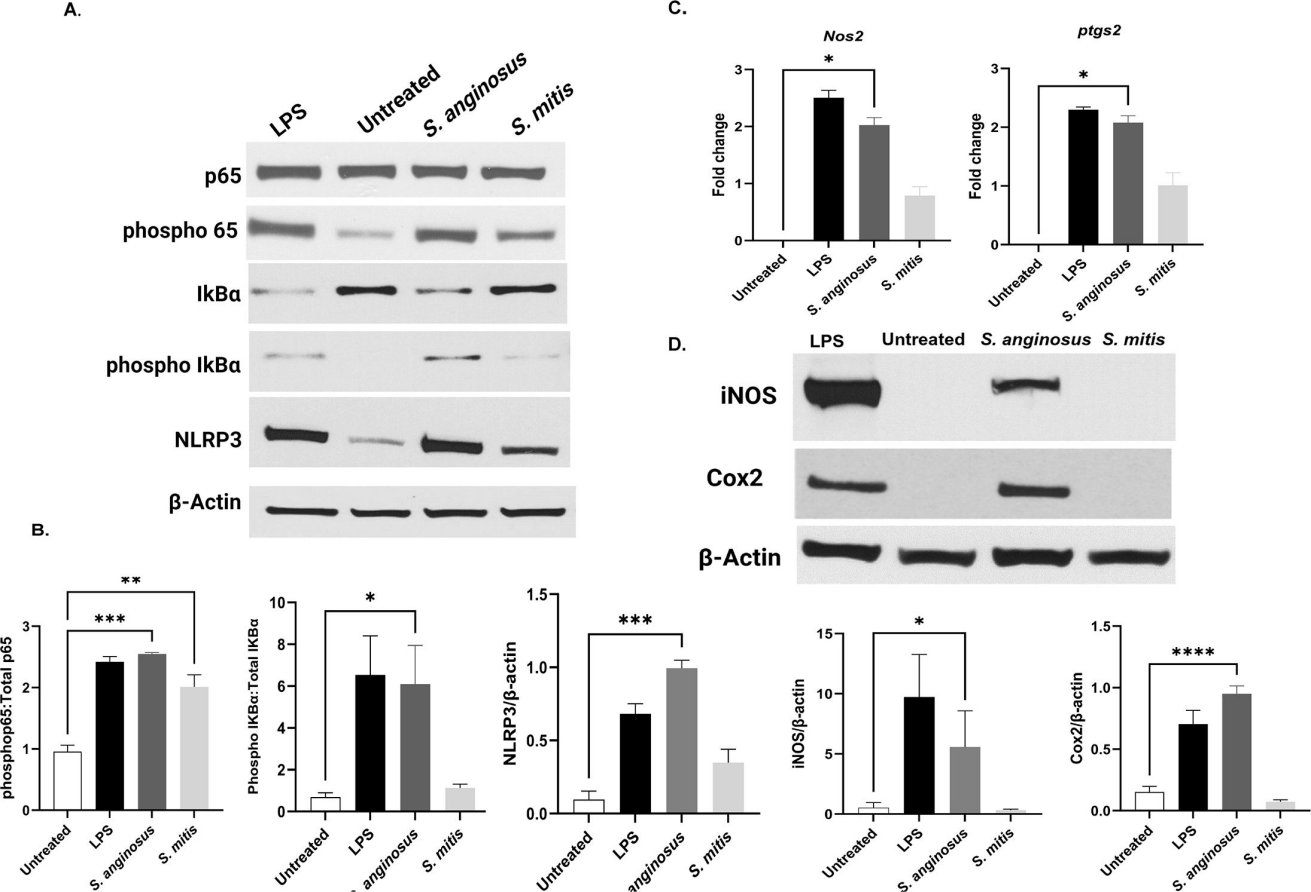
*S. anginosus* infection elicits significant upregulation of NF-κB and inflammatory mediators like NLRP3, iNOS and COX2. (A) Western blot analysis of RAW 264.7 cells treated with LPS, S. anginosus and S. mitis for NF-kB and NLRP3; (B). Densitometry analysis of western blot ; (C) mRNA expression of iNOS and Cox2 and (D) Protein expression of iNOS and Cox2 . The data are presented as Mean ± SEM. * *P* < 0.05 and ***P < 0.0001 compared to untreated RAW 264.7 cells (*n* = 3).

The NF-κB family of transcription factors are critical components of the immune response. NF-κB activation in stimulated cells is mediated by the phosphorylation and degradation of the inhibitory component IκBα, which allows for the release and activation of p65 via phosphorylation. Phospho-p65 then translocations to the nucleus where it acts as a transcriptional factor for several inflammatory cytokine genes, including TNF-α, IL-6, and IL-1β. To determine if *S. anginosus* and *S. mitis* also induce differential activation of NF-κB in macrophages, RAW267.4 macrophages were infected with *S. anginosus* or *S. mitis,* and the protein expression of total and phosphorylated p65 and IκBα were analyzed by western blot. Macrophages infected with *S. anginosus* for 1 h resulted in significantly upregulated NF-κB activation, as evidenced by increased expression of phospho-p65 and phospho-IκBα compared to untreated controls (Fig. 5A and 5B). Moreover, no significant differences in NF-κB activation were observed between untreated macrophages and macrophages infected with *S. mitis* at this time point. These findings corroborated the increase in proinflammatory cytokines in macrophages infected *S. anginosus* compared to *S. mitis* (Fig. 4A and B). The Nucleotide-binding domain and leucine-rich repeat-containing family, pyrin domain containing 3 (NLRP3), has gained attention as a vital facilitator of the innate immune system, playing a critical role in inflammatory responses during both sterile inflammatory insults and microbial infections, including several *Streptococcus* species (33, 34). Compared to untreated control and *S. mitis*-infected macrophages, *S. anginosus* infected macrophages significantly increased NLRP3 protein expression at 6 h post-infection (Fig. 5A and B).

### *S. anginosus,* but not *S. mitis,* induces inflammatory mediators in macrophages including iNOS and Cox2

Inducible nitric oxide synthase (iNOS) and cyclooxygenase 2 (Cox2) are two inducible enzymes that generate inflammatory mediators, including nitric oxide and prostaglandins, respectively. The upregulation of iNOS and Cox2 during inflammation is regulated by the proinflammatory transcription factor NF-κB. Hence, we aimed to investigate if the expression of iNOS and Cox2 are differentially activated by *S. mitis* and *S. anginosus*. Accordingly, RAW264.7 macrophages were exposed to *S. anginosus* or *S. mitis* infection for 6 h, and the expression of iNOS and Cox2 was evaluated using qPCR and immunoblotting. Our results demonstrated that *S. anginosus* infection induced iNOS and Cox2 expression in macrophages (Fig. 5B-D). Conversely, *S. mitis* failed to upregulate the expression of these enzymes, which was consistent with the differential activation of NF-κB between these two species of the genus *Streptococcus*. These observations demonstrate that when exposed to *S. anginosus*, but not *S. mitis*, macrophages mount a coordinated immune response against bacterial infection by increasing nitric oxide and prostaglandin production.

### Significant upregulation of macrophage ACOD1 is observed during *S. anginosus* infection, but not in response to *S. mitis*

**Fig 6:**
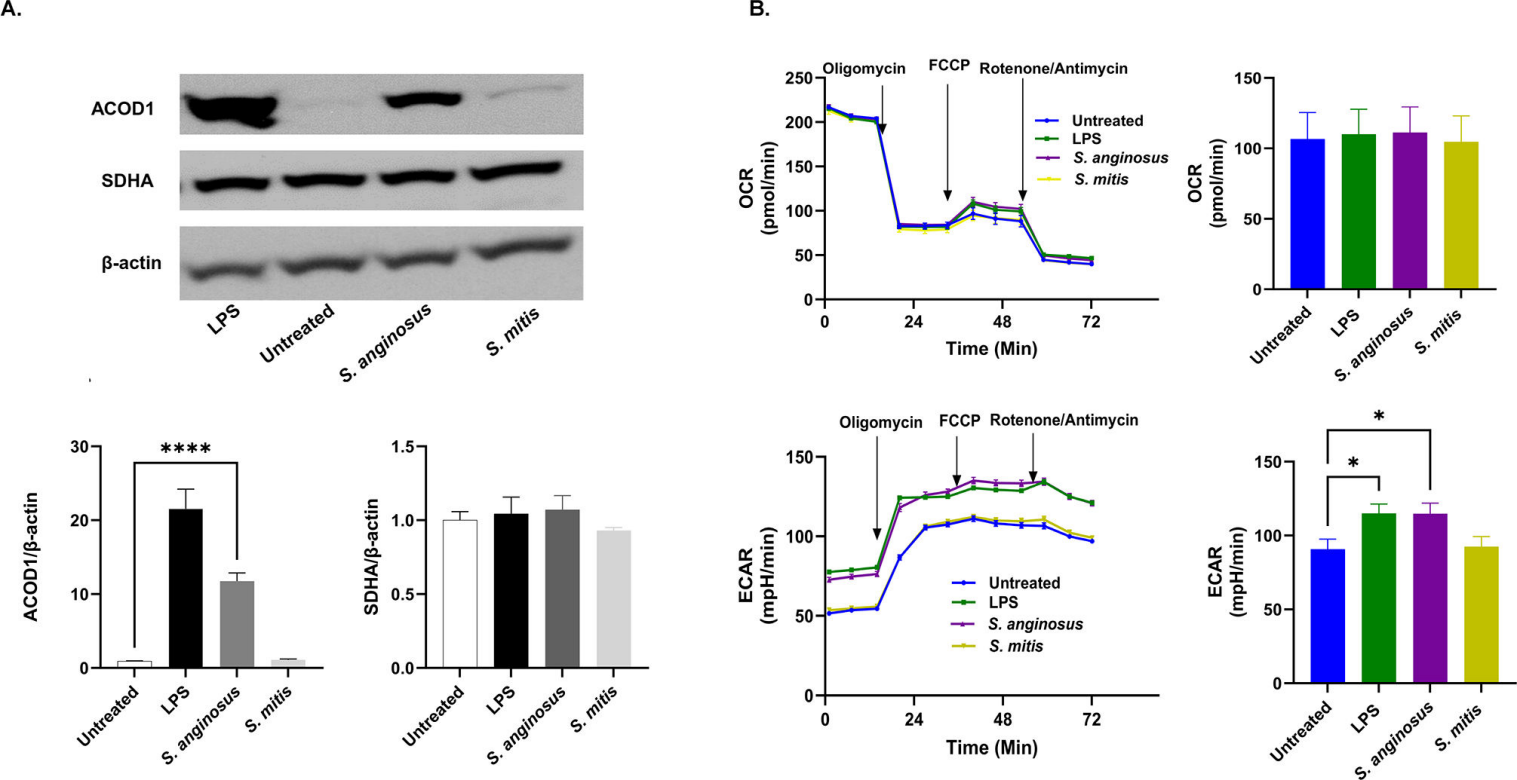
*S. anginosus* induces ACOD1 expression and raises ECAR, distinguishing it from *S. mitis* infection (A) Western blot and densitometry analysis of ACOD1 and SDHA. (B) Seahorse xfe96 analysis of OCR and ECAR. The data are presented as Mean ± SEM. * *P* < 0.05 and ***P < 0.0001 compared to untreated RAW 264.7 cells (*n* = 3).

Inflammation triggers immune cell activation, shifting them from a quiescent state to an effector mode, marked by a significant revamp in cellular metabolism. This metabolic shift involves a profound restructuring of the citric acid cycle, a succession of enzymatic reactions within mitochondria. This cycle is the ultimate metabolic route for oxidizing multiple macronutrients such as carbohydrates, amino acids, and fats. Although the tricarboxylic acid (TCA) cycle supports bioenergetics and biosynthesis, the role of the TCA cycle in macrophage polarization is not limited to ATP production. However, macrophages differentially utilize metabolic pathways to support maximal activation of phenotypes and effector functions (35). Classically activated M1 macrophages exhibit distinct modifications in the TCA cycle, characterized by interruptions after the metabolites citrate and succinate due to the upregulated expression of aconitate decarboxylase (ACOD1/IRG1) and inhibition of SDH enzymes. This metabolic reprogramming generates metabolites like itaconate and succinate, which can exert immunomodulatory effects by influencing gene expression, inflammatory responses, and immune regulation. After assessing NF-κB, iNOS, and Cox2, which are hallmarks of proinflammatory M1 macrophages, we next investigated the protein expression of ACOD1. This metabolic enzyme converts citrate to itaconate, and SDH, which catalyzes the conversion of succinate to fumarate. *S. anginosus* infection significantly upregulated ACOD1 protein expression in macrophages compared to untreated macrophages in 6 h post-infection (Fig. 6A). However, no significant alterations were observed in the expression of SDH in untreated controls or during *S. mitis* infection, suggesting a specific induction of ACOD1 in macrophages during *S. anginosus* infection. This observation signifies a potential role for TCA metabolic reprogramming in the context of *S. anginosus-*induced macrophage activation, which is absent during *S. mitis* exposure.

### *S. anginosus*, but not *S. mitis*-infected macrophages displayed increased ECAR

The measurement of extracellular rates of acidification and oxygen consumption rates provides a powerful way to assess the total energy metabolism of a cell. Oxygen consumption rate (OCR) quantifies how cells consume oxygen during mitochondrial respiration. In contrast, the extracellular acidification rate (ECAR) reflects the rate at which cells release protons (H+) into the extracellular environment, primarily due to the process of glycolysis (36). To assess the influence of *S. mitis* and *S. anginosus* on macrophage immunometabolism and bioenergetics, we evaluated alterations in OCR and ECAR after infecting the macrophages with these oral *Streptococcus* species for 6 h. Our findings revealed that *S. anginosus* triggered heightened macrophage ECAR, while *S. mitis* failed to induce observable changes in macrophage OCR. In contrast, neither *S. anginosus* nor *S. mitis* induced noteworthy variations in OCR, implying that 6 h of infection had minimal impact on mitochondrial respiration and ATP production (Fig. 6B). The increased ECAR, accompanied by a lack of alterations in OCR during *S. anginosus* infection, suggests a potential metabolic shift toward glycolysis induced by *S. anginosus,* which was absent in *S. mitis* infection (Fig. 6B). This finding suggests *S. anginosus* and *S. mitis* have differential capacities to alter macrophage metabolism to drive cellular activation and proinflammatory signatures. Further investigation is required to understand this differential metabolic shift’s underlying mechanisms and functional consequences.

### *S. anginosus* and *S. mitis-*infected macrophages exhibit distinct immune-modulatory metabolite profiles and itaconate-to-succinate ratio

**Fig 7:**
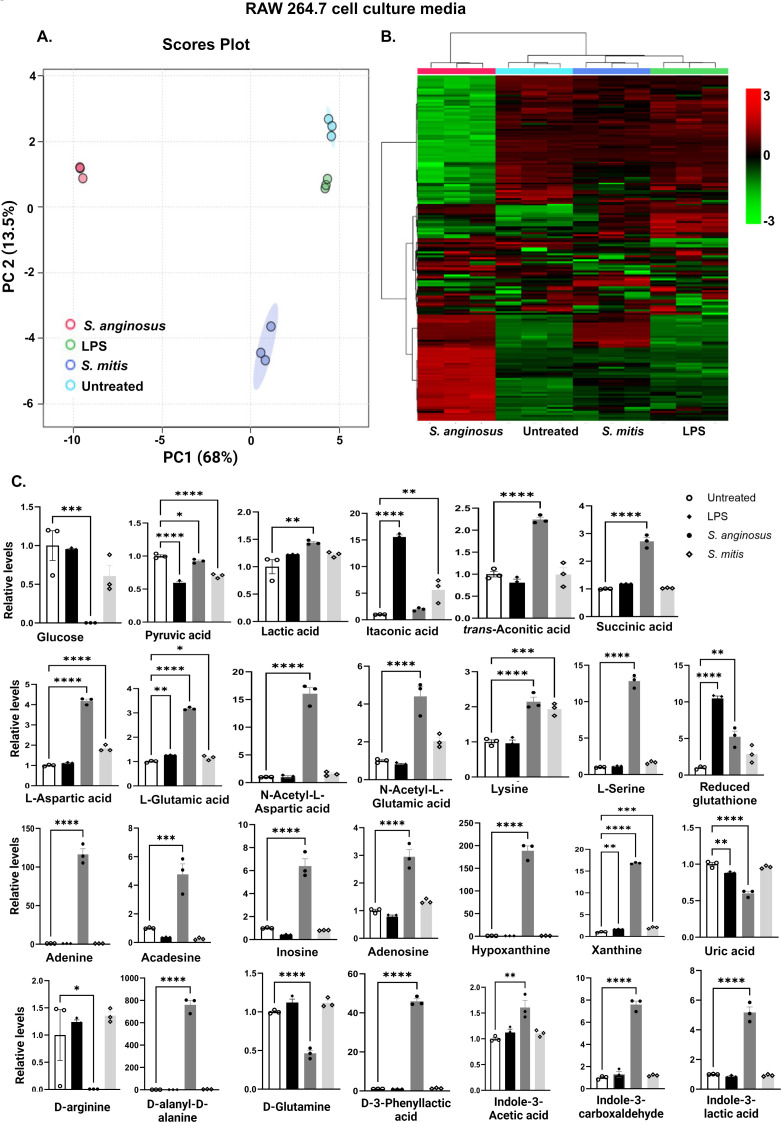
Unique metabolite profile of *S. anginosus*-infected macrophages showcasing heightened expression of various immunomodulatory molecules (A) PCA Score Plot (*n* = 3). (**B**) Heat Map showing metabolite profile of experimental groups with *S. anginosus* displaying distinct metabolites profile. Color gradient: Red to black to green indicates relative metabolite level differences (scale adjacent). (**C**) Bar Graph of critical metabolites related to infection and immunity, Analyzed in GraphPad Prism. Data presented as Mean ± SEM. *P < 0.05 and ***P < 0.0001 compared to untreated RAW 264.7 cells (*n* = 3).

Many bacteria secrete small diffusible signal molecules to sense the local environmental conditions, including their population, and to synchronize multicellular behaviors. While several studies have focused on the intracellular concentration of various metabolites in RAW264.7 cells treated with LPS and other stimuli, we wanted to study the impact of microbial infection on extracellularly released metabolites, which may regulate microbial survival during host interactions. We performed untargeted metabolomics analysis on the supernatants of *S. anginosus* versus *S. mitis*-infected RAW264.7 macrophages and observed a demarcated cluster of metabolites in *S. anginosus* infected cells compared to *S. mitis*-infected or untreated macrophages, as shown by the PCA plot (Fig. 7A) and a unique metabolite profile as shown in the heat map (Fig. 7B). The *S. anginosus* profile differences are highlighted by reduced glucose, arginine, and D-glutamine levels along with elevated lactic acid, amino acids, and other immune-related metabolites like acadesine, uric acid, D-amino acids, D-Phenyl lactic acid and indole metabolites (Fig. 7C).

Notably, the upregulation of specific metabolites such as acetylated lysine and itaconate-to-succinate/trans-aconitic acid ratio modulation demonstrated distinctive immune responses induced by *S. anginosus* and *S. mitis*. Table S1 shows the list of observed metabolites with their respective fold change in different experimental conditions. These findings provide insights into the unique immune-regulatory metabolite interactions triggered by these bacteria during infection, and the question of whether this ratio governs immune stimulation or suppression remains an uncharted domain, along with the potential role of trans-aconitic acid in this transition.

## DISCUSSION

In this study we explored macrophage responses to *S. anginosus* and *S. mitis* and observed distinct immune and metabolic reactions triggered by these *Streptococcus* species. Microscopic examination revealed unique morphological alterations in macrophages infected with *S. anginosus*. Crystal violet staining indicated elongated cell structures (37), while Giemsa staining showcased an increased cytoplasmic area and peripheral nuclei (28). These changes resemble signs of activation, suggesting the potential for *S. anginosus* to prompt distinct immune responses. These morphological changes were largely absent in *S. mitis-*infected macrophages, which provided early observations that the macrophage response to these two bacteria is distinct. It remains to be determined if factors produced by *S. anginosus* and *S. mitis* facilitate the differential proinflammatory response or lack thereof, respectively, during macrophage infection. Streptolysin S (SLS), a peptide hemolysin/cytolysin produced by *S. anginosus*, is linked to cytotoxic effects *in vitro*. β-hemolytic *S. anginosus subsp. anginosus* (SAA) induced cytotoxicity in cocultured HSC-2 cells *in vitro*, suggesting a potential association between *S. anginosus and* SLS (38). However, contradictory findings emerged from Asam et al. (39), which argues SLS produced by SAA fails to induce cytotoxicity in THP-1 cells and human granulocytes. However, in our current study, *S. anginosus* demonstrated no significant cytotoxicity toward RAW267.4 macrophages. Similarly, mitilysin, a hemolysin from *S. mitis*, and related hemolysin, also exhibit cytotoxicity toward THP-1 cells (40), yet no cytotoxic effects were observed during *S. mitis* infection in the present study. These findings suggest other non-cytotoxic factors produced by different *Streptococcus* species may have impact on macrophage inflammatory responses, which necessitates further exploration.

Recognition of PAMPs by TLRs in innate immune cells initiates complicated signaling pathways leading to the activation of several transcription factors including NF-κB (41). *S. anginosus* secretes an antigen termed SAA, a tyrosine tRNA synthetase (TyrRS) affiliated with the aminoacyl-tRNA synthetase family. The precise mechanism underlying SAA secretion remains unknown, as it lacks the conventional N-terminal signal peptide required for recognition by the secretion system (42). SAA has been demonstrated to dose-dependently stimulate NO production and induce the accumulation of NO synthetase mRNA *in vitro* in peritoneal exudate cells (PEC). SAA also induces the accumulation of TNF-α, IL-1β, and IL-6 mRNA (43). These reports correlate with the upregulation of NLRP3, NF-κB, and inflammatory cytokines, including TNFα, IL-6, and IL-1β, observed in *S. anginosus*-infected macrophages in the present study. Unlike *S. anginosus*, *S. mitis* failed to elicit significant activation of NF-κB and downstream targets like iNOS and COX2 in macrophages.

Expanding our investigation, we assessed the influence of these oral streptococci on macrophage polarization and found that both iNOS and Cox2, which are associated with proinflammatory M1 macrophages, were upregulated by *S. anginosus* infection but not in *S. mitis*-infected macrophages. However, *S. anginosus*-infected macrophages also showed increased IL-10 expression, possibly as a compensatory response. Surprisingly, *S. mitis*-infected macrophages did not exhibit this increase in IL-10 or other M2 marker gene expression, ruling out M2 polarization as the reason for *S. mitis*’ subdued M1 response. The absence of proinflammatory M1 or anti-inflammatory M2 responses in *S. mitis*-infected macrophages raises intriguing questions warranting further exploration into its mechanisms of interaction and potential evasion of immune surveillance.

**Fig 8:**
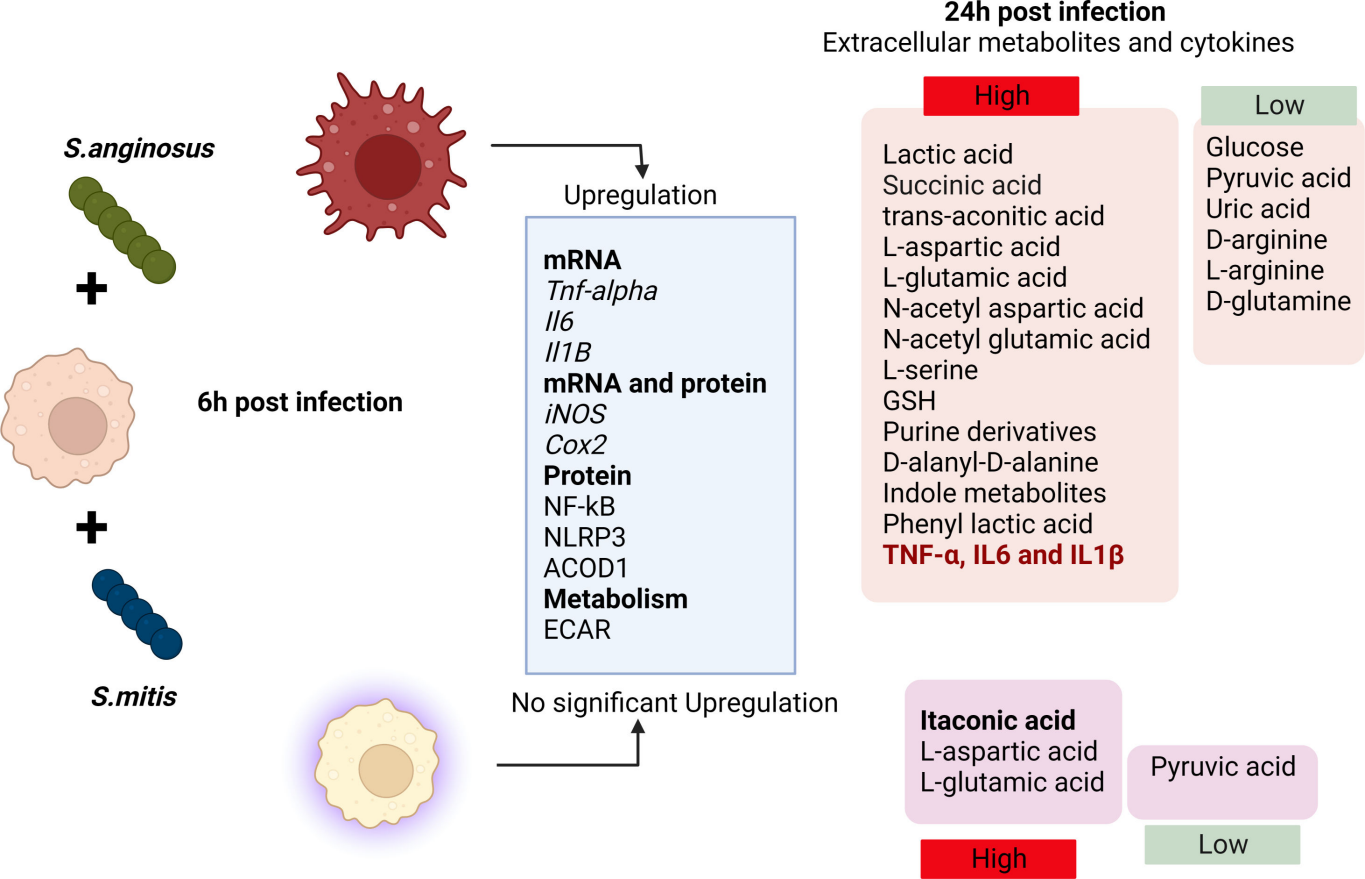
Graphical overview. Infection of macrophages by *S. anginosus* and *S. mitis* displays different morphological, immune, and metabolic changes 6 h and 24 h post-infection.

Immunometabolism is crucial in dictating the outcome of host-pathogen interactions, with macrophage metabolism influencing the capacity to combat infection. Immune cell disruption, which can occur during infection or tissue damage, releases signals from infected, dying, or stressed cells, inducing precise and timely inflammatory responses (44). To explore the bacteria-macrophage metabolic interplay, we conducted non-targeted metabolomics on infected macrophage supernatants to identify secreted metabolites and real-time mito-stress tests for broader metabolic analysis and the overall metabolic profile of macrophages in response to *S. anginosus* and *S. mitis* is outlined in Fig. 8. *S*. *anginosus-*infected macrophages displayed a heightened consumption of glucose and increased production of lactic acid and ECAR. Upregulation of glucose utilization is a hallmark of classical macrophage activation, and increased glucose consumption fuels aerobic glycolysis, a consequence of diverting pyruvate (a product of glucose metabolism in the cytosol) toward lactate production rather than oxidation in the mitochondrial TCA cycle (45). Following the detection of bacteria, macrophages switch their metabolism from oxidative respiration through the tricarboxylic acid cycle to high-rate aerobic glycolysis to meet the increased energy demands necessary to synthesize inflammatory cytokines (46, 47). However, macrophage OCR and ATP production among the *Streptococcus*-infected and control macrophages did not differ significantly, suggesting that mitochondrial respiration is unaffected in any tested conditions at our experimental time point.

In this study, both *S. anginosus* and *S. mitis* significantly decreased macrophage extracellular pyruvate, a metabolite that enters diversified pathways in adaptation to cellular needs (48). *S. anginosus* induced a higher conversion of macrophage pyruvate to lactate in comparison to *S. mitis*. Alternatively, in *S. mitis* infection, pyruvate might be channeled toward the TCA cycle, as we detected an increase in itaconate in media from *S. mitis*-infected macrophages but not from macrophages infected with *S. anginosus* for 24 h. This redirection could potentially enhance NADH generation, fueling the oxidative phosphorylation observed in resting cells. Glucose levels remained stable in *S. mitis*-treated macrophages, consistent with the findings of Newsholme et al. (49) who reported a low rate of glucose utilization by “resting” macrophages *in vitro*, with a sharp increase in glycolysis during phagocytosis or heightened secretory activity. Sun et al. (50) demonstrated that the impact of particulate matter on mitochondrial oxygen consumption varies with time, suggesting that exploring metabolic flux over more extended incubation periods could modify the OCR. This avenue is worth further investigation, especially regarding *S. mitis* versus *S. anginosus* infection.

The Warburg effect, marked by impaired mitochondrial respiration, is typically linked to M1 proinflammatory macrophages, often accompanied by a disrupted mitochondrial citric acid cycle (TCA cycle). It is proposed that activated macrophages downregulate the mRNA levels of the enzyme isocitrate dehydrogenase (IDH), creating a cellular pool of citrate that supports fatty acid synthesis and the generation of lipid intermediates like prostaglandins that promote inflammation. Decreases in IDH activity may also contribute to the accumulation of citrate-related metabolites, supporting the production of itaconate (51), a metabolic byproduct derived from cis-aconitic acid in the TCA cycle, which is catalyzed by cis-aconitate decarboxylase (IRG1/CAD/ACOD1). In proinflammatory cells, itaconate serves two critical functions. First, it inhibits bacterial isocitrate lyase (52), disrupting the glyoxylate shunt, a critical metabolic pathway that impairs bacterial growth in carbohydrate-limiting conditions. Secondly, it inhibits SDH, an enzyme that converts succinate to fumarate within the host cell, leading to succinate accumulation (53, 54). *S. anginosus* infection exhibited temporal dynamics on ACOD1 expression (upregulation following 6 h post-infection) and Itaconate production (no significant increase in 24 h post-infection). Further, *S. anginosus* infection also causes a surge in the TCA cycle metabolite succinate and trans-aconitic acid an isomer of cis-aconitate and a precursor for itaconate reported to be found in *Pseudomonas* (55) and certain plants (56). While succinate accumulation and itaconate accumulation are hallmarks of proinflammatory macrophages, we found elevated extracellular itaconate in *S. mitis-infected* macrophages at 24 h, which differed from *S. anginosus* infection that might exert differential inflammatory responses.

Succinate, an intermediate metabolite of the TCA cycle, has the potential to accumulate under specific conditions such as inflammation and stress. It is widely produced by different tissue and immune cells, and it can contribute to inflammation and stress-induced immune responses. Microbial disruptions in gut microbiota metabolism and cross-feeding can lead to succinate accumulation, especially with dietary changes, indigestible carbohydrate consumption, and antibiotic use, resulting in elevated fecal succinate levels in the elderly and various animal models as reviewed by Connors et al. (57). In combination with LPS stimulation, succinate enhances HIF-1α stability, driving IL-1β transcription (58). When released extracellularly, succinate signals through upregulated SUCNR1/GPR91 and magnifies IL-1β production, establishing a macrophage activation loop (59). Conversely, studies using SUCNR1-deficient peritoneal macrophages revealed similar LPS-induced cytokine profiles as wild-type controls (60). Moreover, Keiran et al. (61) demonstrated that succinate/SUCNR1 signaling promotes an anti-inflammatory macrophage phenotype, emphasizing succinate’s dual role as a proinflammatory danger signal and stress-induced alarmin alerting the immune system. The source and impact of elevated extracellular succinate in macrophages in response to *S. anginosus* infection warrants further investigation.

L-Arginine (L-arg) is a versatile amino acid and a central intestinal metabolite in mammalian and microbial organisms and serves as a substrate for intestinal and microbial cells that colonize the largest interface at which our body communicates to the microbiota. L-arg deprivation prolongs bacterial persistence and perpetuates chronic inflammation (62). D-Glutamine, an enantiomer of the conditionally essential amino acid L-glutamine, is reduced in the serum of rats in a model of acute pancreatitis (63) and in patients with hepatocellular carcinoma (64). *S. anginosus-*infected macrophages displayed significantly decreased levels of D-glutamine and L arginine, suggesting S. *anginosus* has a greater capacity to utilize these amino acids compared with *S. mitis*, which may contribute to S. *anginosus* pathogenicity. These novel findings warrant further investigation to better understand how *S. anginosus* mediates immune responses in the context of oral biology and squamous cell carcinoma progression. Microbiota-derived small molecules, like indole and its derivatives (65), along with phenyl lactic acid (66), have been identified for their intricate interaction with the host, showcasing a diverse array of localized and heterotopic biological effects. Notably, their expression is heightened during *S. anginosus* infection but remains unaltered in *S. mitis*, highlighting a distinct pattern of regulation based on the infecting species.

In summary, here, we found that *S. anginosus* triggers a proinflammatory response at 6 h post-infection yet induces production of IL-10 as well as a distinct extracellular metabolite profile as compared with untreated and *S. mitis* infection. These distinct inflammatory and metabolic shifts in macrophages during *S. anginosus* and *S. mitis* infection warrant further investigation. Considering the lowered extracellular levels of itaconic acid and higher levels of succinic acid induced during *S. anginosus* infection, *S. anginosus* may influence macrophage immunometabolism through the itaconate-to-succinate ratio. This warrants further exploration using primary cells to evaluate the impact of *S. anginosus* and *S. mitis* on these distinct metabolic pathways during inflammatory signaling and immune activation.

## SUPPLEMENTAL MATERIAL

Supplemental material iai.00536-23-s0001.docx Fig. S1 to S3 and Table S1.

ASM does not own the copyrights to Supplemental Material that may be linked to, or accessed through, an article. The authors have granted ASM a non-exclusive, world-wide license to publish the Supplemental Material files. Please contact the corresponding author directly for reuse.
