# Supplementary material for: Oral streptococci S. anginosus and S. mitis induce distinct morphological, inflammatory, and metabolic signatures in macrophages
Source: Infect Immun. 2024 Jan 30;92(3):e00536-23. doi: 10.1128/iai.00536-23 (PMC10929413; doi:10.1128/iai.00536-23)
Supplement: Supplemental material — Fig. S1 to S3 and Table S1. [file iai.00536-23-s0001.docx]

**Supplementary Fig.1: CFU determination for *S. anginosus* and *S. mitis***


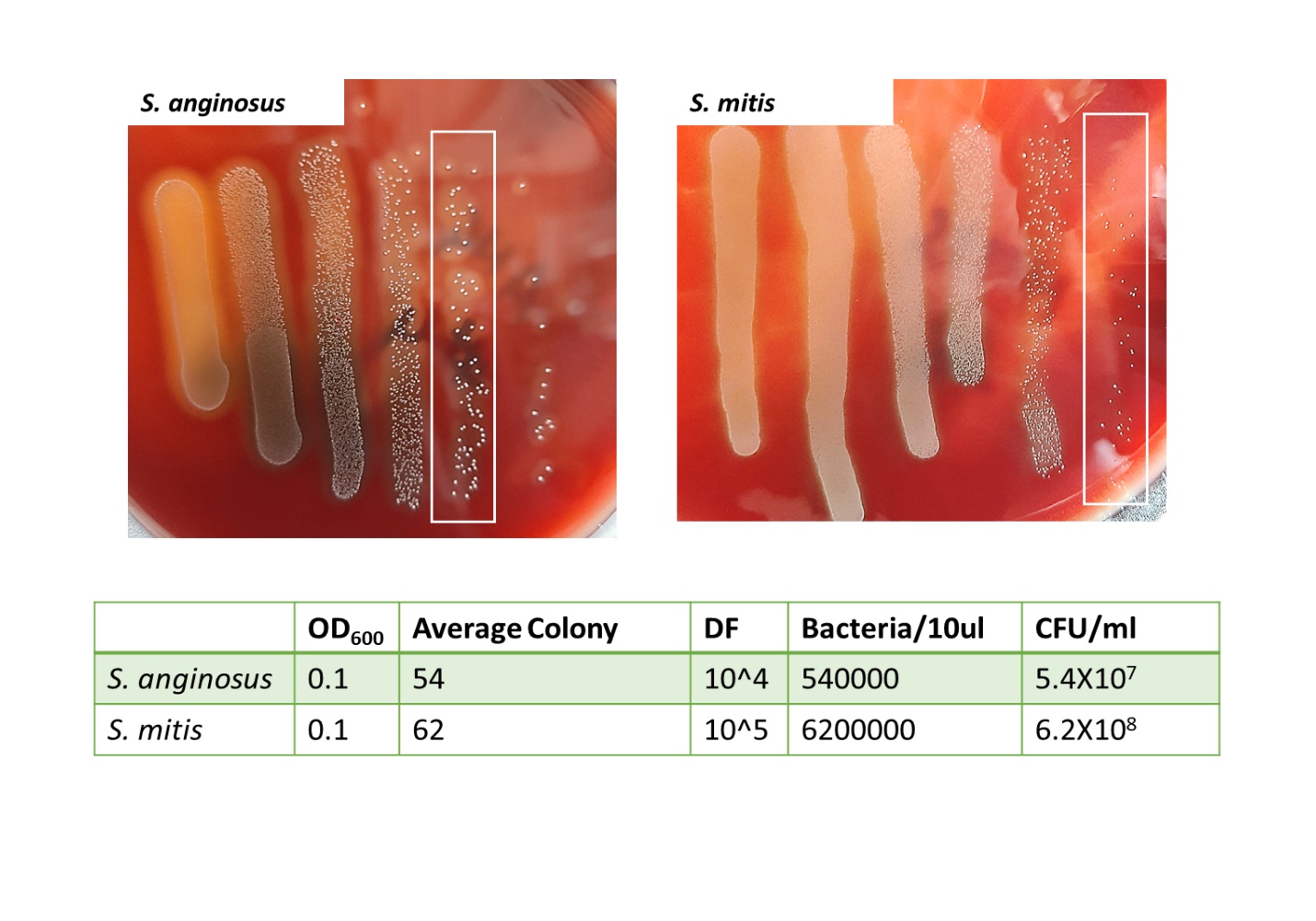


**Supplementary figure 2. Macroscopic and Microscopic observation of RAW264.7 macrophages treated with LPS or *S. anginosus* and *S. mitis* at different MOI and time points.**


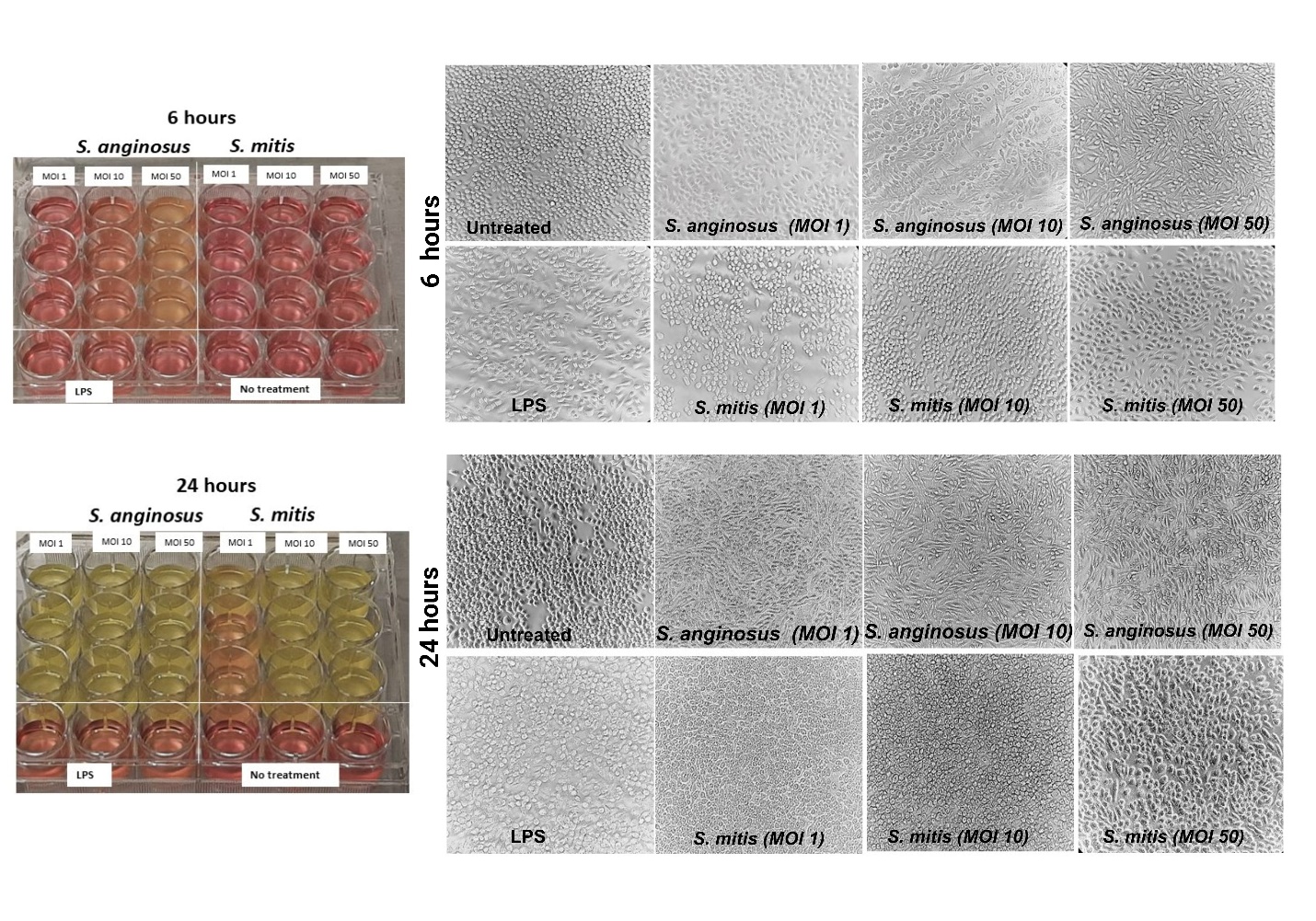


**Supplementary Table 1: Fold change of extracellular metabolites from RAW 264.7 cells in different treatment conditions**

| S.No | Extracellular metabolite | Untreated | LPS | *S. anginosus* | *S. mitis* |
| --- | --- | --- | --- | --- | --- |
| 1 | D-(+)-Glucose | 1 | 0.95 | 0.00 | 0.61 |
| 2 | Pyruvic acid | 1 | 0.59 | 0.92 | 0.70 |
| 3 | L-(+)-Lactic acid | 1 | 1.22 | 1.44 | 1.22 |
| 4 | trans-Aconitic acid | 1 | 0.81 | 2.24 | 0.99 |
| 5 | Itaconic acid | 1 | 15.57 | 1.97 | 5.62 |
| 6 | Succinic acid | 1 | 1.18 | 2.72 | 1.03 |
| 7 | Adenine | 1 | 0.96 | 116.45 | 0.99 |
| 8 | Adenosine | 1 | 0.78 | 2.95 | 1.34 |
| 9 | Inosine | 1 | 0.41 | 6.40 | 0.81 |
| 10 | Hypoxanthine | 1 | 0.95 | 188.69 | 1.09 |
| 11 | Xanthine | 1 | 1.69 | 16.82 | 2.02 |
| 12 | Uric acid | 1 | 0.88 | 0.60 | 0.96 |
| 13 | 2'-Deoxycytidine | 1 | 0.44 | 0.02 | 0.57 |
| 14 | 1-Methylguanine | 1 | 0.88 | 2.89 | 1.07 |
| 15 | 2'-O-Methylguanosine | 1 | 0.94 | 0.84 | 1.10 |
| 16 | 2'-O-Methylinosine | 1 | 0.93 | 0.50 | 1.02 |
| 17 | Betaine | 1 | 4.03 | 5.51 | 1.03 |
| 18 | Choline | 1 | 0.85 | 1.30 | 0.87 |
| 19 | Crotonic acid | 1 | 17.09 | 2.07 | 5.95 |
| 20 | L-Glutathione reduced | 1 | 10.47 | 5.23 | 2.87 |
| 21 | Acadesine | 1 | 0.36 | 4.77 | 0.25 |
| 22 | L-Carnitine | 1 | 1.07 | 1.81 | 1.19 |
| 23 | L-Carnosine | 1 | 0.58 | 0.51 | 0.59 |
| 24 | 15(R)-Prostaglandin D2 | 1 | 65.38 | 63.04 | 52.05 |
| 25 | 15-Deoxy-Δ12,14-prostaglandin D2 | 1 | 29.73 | 47.92 | 22.71 |
| 26 | L-Alanine | 1 | 0.79 | 0.27 | 1.02 |
| 27 | L-Glutamic acid | 1 | 1.26 | 3.18 | 1.16 |
| 28 | L-Arginine | 1 | 0.91 | 0.00 | 1.04 |
| 29 | L-Aspartic acid | 1 | 1.11 | 4.18 | 1.84 |
| 30 | L-Citrulline | 1 | 3.29 | 0.87 | 1.85 |
| 31 | Ornithine | 1 | 4.70 | 2.12 | 2.33 |
| 32 | L-Homoarginine | 1 | 0.88 | 0.67 | 0.86 |
| 33 | L-Phenylalanine | 1 | 0.96 | 1.23 | 1.02 |
| 34 | L-Tyrosine | 1 | 0.95 | 0.99 | 0.99 |
| 35 | 1H-Imidazole-1-acetic acid | 1 | 0.95 | 3.19 | 0.96 |
| 36 | L-Serine | 1 | 1.12 | 12.82 | 1.65 |
| 37 | L-Histidine | 1 | 0.91 | 0.56 | 0.96 |
| 38 | N-Acetylglutamic acid | 1 | 0.81 | 4.40 | 2.04 |
| 39 | N-Acetyl-L-Lysine | 1 | 0.96 | 2.15 | 1.94 |
| 40 | N6-Acetyl-L-lysine | 1 | 0.95 | 1.26 | 1.34 |
| 41 | N-Acetyl-L-aspartic acid | 1 | 1.01 | 16.04 | 1.56 |
| 42 | L-Tryptophan | 1 | 0.98 | 1.16 | 1.06 |
| 43 | DL-Kynurenine | 1 | 0.99 | 1.24 | 1.08 |
| 44 | Indole-3-acetic acid | 1 | 1.12 | 1.61 | 1.11 |
| 45 | 5-Hydroxyindole-3-acetic acid | 1 | 1.36 | 0.42 | 1.30 |
| 46 | Indole-3-carboxaldehyde | 1 | 1.29 | 7.60 | 1.20 |
| 47 | Indole-3-lactic acid | 1 | 0.85 | 5.17 | 0.93 |
| 48 | Allantoin | 1 | 0.96 | 0.90 | 0.95 |
| 49 | D-(+)-Tryptophan | 1 | 0.97 | 1.61 | 1.03 |
| 50 | D-3-Phenyllactic acid | 1 | 1.04 | 45.90 | 1.32 |
| 51 | D-Alanyl-D-alanine | 1 | 1.19 | 760.62 | 4.81 |
| 52 | D-Arginine | 1 | 1.24 | 0.01 | 1.36 |
| 53 | D-Aspartic acid | 1 | 1.19 | 14.03 | 2.25 |
| 54 | D-Glutamic acid | 1 | 1.15 | 1.23 | 1.16 |
| 55 | D-Glutamine | 1 | 1.12 | 0.46 | 1.12 |
| 56 | D-Histidine | 1 | 0.87 | 1.13 | 0.88 |
| 57 | L-Phenylalanine | 1 | 0.96 | 1.23 | 1.02 |
| 58 | D-(-)-Quinic acid | 1 | 0.64 | 0.38 | 0.67 |
